# Direct Measurements of Oxygen Gradients in Spheroid Culture System Using Electron Parametric Resonance Oximetry

**DOI:** 10.1371/journal.pone.0149492

**Published:** 2016-02-22

**Authors:** Laura M. Langan, Nicholas J. F. Dodd, Stewart F. Owen, Wendy M. Purcell, Simon K. Jackson, Awadhesh N. Jha

**Affiliations:** 1 School of Biological Sciences, Plymouth University, Plymouth, PL4 8AA, United Kingdom; 2 School of Biomedical & Healthcare Science, Plymouth University, Plymouth, PL4 8AA, United Kingdom; 3 AstraZeneca, Alderley Park, Macclesfield, Cheshire, SK10 4TF, United Kingdom; Martin-Luther-Universität Halle-Wittenberg, GERMANY

## Abstract

Advanced *in vitro* culture from tissues of different origin includes three-dimensional (3D) organoid micro structures that may mimic conditions *in vivo*. One example of simple 3D culture is spheroids; ball shaped structures typically used as liver and tumour models. Oxygen is critically important in physiological processes, but is difficult to quantify in 3D culture: and the question arises, how small does a spheroid have to be to have minimal micro-environment formation? This question is of particular importance in the growing field of 3D based models for toxicological assessment. Here, we describe a simple non-invasive approach modified for the quantitative measurement and subsequent evaluation of oxygen gradients in spheroids developed from a non-malignant fish cell line (i.e. RTG-2 cells) using Electron Paramagnetic Resonance (EPR) oximetry. Sonication of the paramagnetic probe Lithium phthalocyanine (LiPc) allows for incorporation of probe particulates into spheroid during its formation. Spectra signal strength after incorporation of probe into spheroid indicated that a volume of 20 μl of probe (stock solution: 0.10 mg/mL) is sufficient to provide a strong spectra across a range of spheroid sizes. The addition of non-toxic probes (that do not produce or consume oxygen) report on oxygen diffusion throughout the spheroid as a function of size. We provide evidence supporting the use of this model over a range of initial cell seeding densities and spheroid sizes with the production of oxygen distribution as a function of these parameters. In our spheroid model, lower cell seeding densities (∼2,500 cells/spheroid) and absolute size (118±32 *μ*m) allow control of factors such as pre-existing stresses (e.g. ∼ 2% normoxic/hypoxic interface) for more accurate measurement of treatment response. The applied methodology provides an elegant, widely applicable approach to directly characterize spheroid (and other organoid) cultures in biomedical and toxicological research.

## Introduction

Over the past three decades, the use of three dimensional cell culture (e.g. spheroids) has gained increased recognition as an important tool in biological research and in preclinical trials [1–3] over conventional organs or *ex vivo* cultures which are unsurprisingly in short supply. Spheroids are possibly the simplest 3D tissue model in research with arguably the best physiological representation of the native tissue in comparison to other commonly used models such as cells grown as monolayers, tissue slices or *ex vivo* organs. Typically round or elliptic, the spheroids are globe like compact structures which can be manipulated without causing mechanical dissociation of the cells [1]. They are formed through the adherence of cells to one another in preference to a substrate [3–5]. There is an enormous body of literature on spheroid models and their use in cancer therapy orientated studies (3D tumour models) to bridge the gap between cell based assays and *in vivo* studies [6–8]. These systems can be used to model many characteristics of avascular tumours and micrometastases of large solid tumours, in addition to better replicating the barrier to drug penetration represented by native tumour tissue [9]. Previous research has also demonstrated the suitability of the spheroid system as an *in vitro* alternative to the assessment of chemical toxicity and evaluation of environmental samples in biological and ecotoxicological studies [1, 2, 10–12]. However, in order to use such a 3D system in non tumour models, we need to understand more about the mass transport limitations of the non-tumour model, especially in respect of oxygen transport.

To our knowledge, there has been no attempt to directly measure oxygen consumption or quantify oxygen micro-environment formation non destructively in spheroid based models (both tumour based and non tumour derived) until the present study. However, it should be noted that although these questions have not been directly addressed in the literature, EPR has previously been used as a measure of cytotoxic response to a toxic drug [13]. Micro-environment formation in spheroids involves the metabolic adaptation of cells in response to this new environmental structure (e.g. from monolayer to suspension culture) and can cover changes in lactate accumulation, glucose distribution, cellular proliferation and the response of cells to external stresses such as diffusive gradients (e.g. oxygen) [14]. The availability of oxygen in tumour spheroid systems is critical for metabolism, in addition to controlling the responsiveness to experimental drug treatments [5, 14, 15]. The formation of micro-environments within spheroids growing under *in vitro* conditions is determined by the balance between oxygen diffusion from the growth medium and its consumption within the spheroid. While this relationship has been well established in tumour biology [16–18], there appears to be no studies addressing this directly in non-tumour models.

Previously, the formation of oxygen micro-environments in tumour spheroid models has been estimated using a two-pronged approach. One method defines the micro-environment boundary in the spheroid model (for example, oxygen-sensitive probes such as the Whalen type electrode or Clark electrode [18–20]). Following this the data is fed into a pre-established differential equation of diffusion under consideration of the previously defined micro-environment boundary [21]. Alternatively, mathematical modelling of spheroid micro-environments is feasible with some basic information of the spheroid obtained destructively through histochemical staining of spheroid sections [22]. It should be noted that there is little consensus in the literature on the most appropriate method to use due to recently documented difficulties/limitations of these methods [23].

The use of spin-label oximetry (with paramagnetic probes) to biological systems dates back over 40 years [24, 25]. Electron Paramagnetic Resonance (EPR alternatively known as ESR) oximetry has wide applications in biomedical research [26] and oximetry represents a small subset of this broad field. EPR oximetry is a relatively simple, non-invasive method to measure oxygen levels in biological systems using implanted and soluble paramagnetic probes, the use of which has been gradually increasing [27–30]. The method is based on the Heisenberg spin exchange between paramagnetic molecules of probe and oxygen causing a change in linewidth of the EPR spectrum of the probe [27, 31, 32]. One of the most important characteristics of this approach is that it does not interfere with oxygen metabolism within the biological system, therefore providing a basis for non-invasive oxygen measurements in biological systems [33], a critical requirement for measurement of oxygen within 3D models [29].

**Fig 1:**
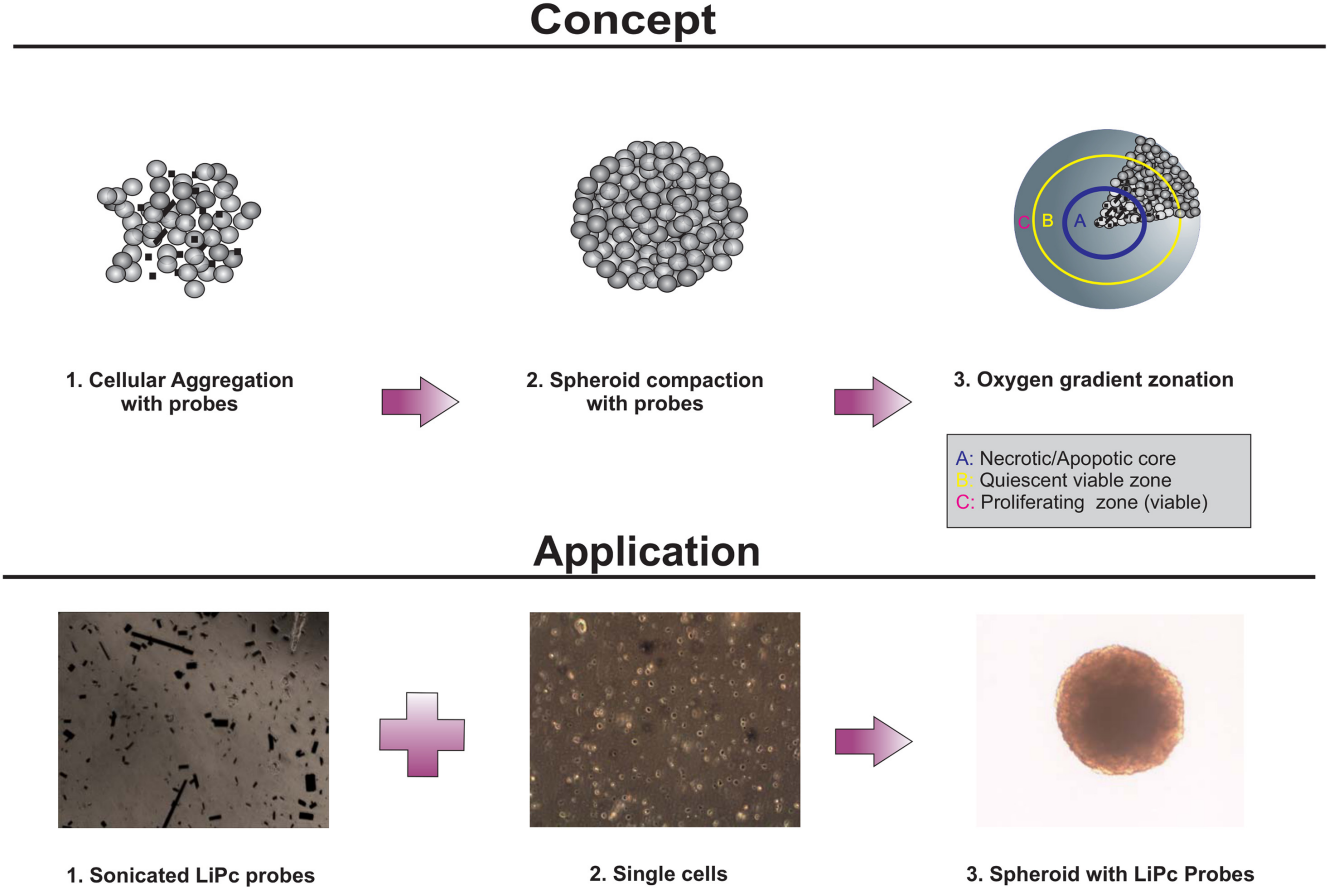
Moving from experimental concept to application. Overall experimental design to investigate the feasibility of moving from theoretical concept to application of EPR oximetry to the spheroid model. The incorporation of paramagnetic probes into the model will allow for the non-invasive determination of oxygen content/micro-environment.

In the present study, we move from concept to application (Fig 1) by first establishing that spheroids could form around paramagnetic probe particulates (S1 Fig). Following this establishment, we then used EPR oximetry to identify variations in oxygen concentration levels as a function of spheroid size and at different time points. Finally, micro-environments within the model system at various times and spheroid sizes were formed (as above). The results obtained were extended further through the application of mathematical formulae to identify zones of senescence/necrosis [22]. The results provide the evidence required to show maximum spheroid size to be used in areas of research requiring the absence/minimal necrosis in the spheroid system.

## Methods

### Reagents

All chemicals and reagents were purchased from Life Technologies (UK) and Sigma Aldrich (UK). The paramagnetic probe Lithium phthalocyanine (LiPc) was a kind gift from Dr. Philip James, Cardiff University School of Medicine, UK. Paramagnetic probes are also available commercially (for example Alfa Aesar, a Johnson Matthey Company, Massachusetts, USA).

### Preparation of paramagnetic probes

Initially, LiPc was ground using a marble morter and pestle in Dulbecco’s phosphate buffered saline (DPBS) to make up a 1mg/mL stock solution. From this a 0.1mg/mL solution was chosen as the optimum probe strength to use in combination with the spheroids after optimisation of spectra signal strength and intensity via EPR. In order to reduce the size of the probes to allow for incorporation into the spheroids, the chosen paramagnetic probe was sonicated in unconditioned DPBS at 4°C in an Elmasonic S15 ultrasonic washer (37 kHz; ELMA Hans Schmidbauer, Germany). As previously reported, paramagnetic probes become smaller with longer sonication duration [34]. After exploring various optimisation regimes (no sonication versus varying sonication times of 1–5 h), a final sonication time of 5 h was determined to yield sufficiently small probe particulates measured as per size measurements section below (11.20 ± 1.02 *μ*m). Particulates of this size were easily incorporated during the formation of the spheroid.

### Monolayer culture

Unlike mammalian tumour cell lines which have been well studied in our group [35, 36], with well defined micro-environments (see [10]), little information exists on spheroids derived from non-tumour sources in terms of spheroid heterogeneity. The rainbow trout (*Oncorhynchus mykiss*) gonad cell line RTG-2 [37] was established from *Oncorhynchus mykiss* gonads and is being widely used in the field of *in vitro* toxicology due to its reproducible results in inter-laboratory validations of cytotoxicity, capacity for metabolism of xenobiotics and well characterised protein synthesis and oxygen consumption [38–40]. This fibroblastic cell line was obtained from the ECACC (European Collection of Cell Cultures, Public Health England; ATCC CCL 55) and is routinely used in our laboratory under standard culture conditions [41, 42]. Briefly, the cells were cultured in 75cm cell culture flasks (Greiner, UK) in Minimal essential medium (MEM) supplemented with Non-essential Amino Acids (NEAA), 2mM L-glutamine, 10% fetal bovine serum (FBS), gentimicin (10*μ*g/mL) and 5% CO_2_ in a 19°C incubator. Cell proliferation was maintained by weekly sub-cultivation (split 1:3) or seeding at 5×10^4^ cells/mL [41, 42].

### Validation of spheroid formation with paramagnetic probe

Confluent cultures of RTG-2 cells were trypsinized and cell number counted and transferred at defined seeding densities to non-tissue culture treated 96-well u-shaped micro-plates (Greiner, UK, Cat-No. 650-180) that had been pre-coated with 60*μ*L of a 0.6% of Poly(2-hydroxyethyl methacrylate)(pHEMA) solution (P3932, Sigma) (dried for 48–56 h in a sterile culture cabinet at 37°C with lids on) to minimise cellular attachment. To the seeded wells, 20*μ*L of 0.1mg/mL of the paramagnetic probe LiPc was added. The 96 well plate was gassed with 5% CO_2_ and placed at 19°C in a refrigerated incubator (New Brunswick Galaxy 170R, Eppendorf) on an orbital shaking platform at a constant rotation speed of 83 rpm. After 24 h, when aggregates of cells encasing the probes had formed, the rotation speed was reduced to 80 rpm. The culture media was exchanged every three days after initial seeding, by exchanging 100*μ*L old media for 100*μ*L fresh media. During plating up, 200*μ*L of PBS was added to the peripheral wells of the 96 well tissue culture microtiter plate in order to maximise spheroid development and recovery [43]. Cellular aggregates were visible after 24h, but did not form spheroids until day 7, as indicated by a plateau in spheroid volume (see section on Size Measurements). Following verification of spheroid formation with sonicated LiPc (S1 Fig), the RTG-2 cells were seeded at a range of densities in order to validate the appropriateness of the methodology for a range of spheroid sizes. Oxygen concentration is presented as a percentage of fully oxygenated probes and makes the following assumptions: (a) the spheroid is spheroidal in structure allowing even uptake of oxygen over the whole model, and (b) oxygen is calculated as a percentage of all probe particulates within the model (linewidth) irrespective of probe location (heterogeneous distribution due to particulate size).

#### Size measurements

The RTG-2 single cell size ranges from 11 ±3*μ*m after trypsin treatment, however it is known that cell size is dependent on stage of growth differentiation and attachment to substrate (for example, [44]). In order to measure variation in size of spheroids, diameters of a selection of spheroids at different seeding densities (5 seeding densities: 2,500-80,000 cells/spheroid as per Table 1) were recorded. Biological replicates were defined as non-parallel passages, with each size measurement of all seeding densities recorded over 3 passages. Each measurement consisted of 8 technical replicates per seeding density and passage. Diameters/radius were calculated from digital images acquired using a microscope mounted digital camera attached to an inverted light microscope (OptixCam, The Microscope Store, USA). Digital images were analysed using OCView7.

**Table 1:** Physiological differences between spheroids of varying sizes at separate sampling times. Oxygen concentration over time (Δ % O_2_) within the spheroids is reported as individual linewidth of the spheroids as a percentage of the fully oxygenated probe and media linewidth. The viable rim of the spheroid, where oxygen is not limited, is calculated based on the total oxygen measurable in the spheroid. The hypoxic zone, where oxygen is limited, is calculated based on the determination of the viable rim, the refinement of which allows for the quantification of the size of the senescent zone within the RTG-2 spheroid. Results are presented as the average of three individual experiments.

Seeding (cells)	Sampling (day)	Δ O_2_ (%)	Radius (*μ*m)	Viable rim (*μ*m)	Hypoxic zone (*μ*m)
2,500	7	88	67 ± 32	59	8
	14	45		30	37
10,000	7	48	200 ± 47	96	104
	14	24		48	152
20,000	7	34	225 ± 43	77	148
	14	27		61	164
60,000	7	28	300 ± 62	84	216
	14	45		135	165
80,000	7	22	350 ± 117	77	273
	14	26		91	259

### EPR Oximetry

All spectra were recorded on a Bruker emx micro EPR spectrometer fitted with variable temperature accessory, operating at 9.4GHz. The sub-lite-wall PTFE (polytetrafluorethylene) tubing (o.d = 0.97mm and i.d = 0.8mm)(Zeuss, Orangeburg, Sc, USA) used during the study of EPR linewidth was placed into quartz EPR tubes open at both ends, and the samples maintained at 292°K (19°C) by a flow of air. The linewidths of the spectra from aqueous suspensions of LiPc in PBS were measured at various temperatures between 292°K and 316°K and the known oxygen solubility at the specific temperatures being used to form a calibration curve. Spectra were also recorded in water, under an atmosphere of nitrogen to obtain the zero oxygen point. Field modulation amplitude (ma) was carefully adjusted depending on EPR linewidth to prevent over-modulation and artificial broadening. Spheroids were drawn into the PTFE gas permeable tubing with ∼5*μ*L of media per spheroid (2 spheroids per linewidth measurement). The tube was folded once, and the spheroids allowed to sediment at the fold. Samples were maintained in the cavity at 292°K, which is equivalent to the incubation temperature. Oxygen concentration was quantified by measuring the peak to peak line width of the spectrum relative to a sample which contained only the probe and media but not spheroids/cells. An example of the broad initial spectra at the initial time point, and the significant narrowing is demonstrated in the supporting information (S2 Fig).

### Determination of Oxygen consumption rate (OCR)

Oxygen consumption rate (OCR) using EPR were obtained for spheroids formed from the RTG-2 cell line, where *μ*mole (*μ*M) of oxygen was measured in a closed chamber over time, as previously described in detail [16, 45]. Briefly, spheroids which had already formed around LiPc (7 days) were drawn into a glass capillary tube with ∼5*μ*L of media per spheroid (n = 5) and sealed at both ends using melted paraffin avoiding the entrapment of any air bubbles. The capillary tube was visually checked for air bubbles and was discarded if it did not conform. Following inspection, the tube was quickly placed inside the microwave cavity and EPR spectra acquisition of the LiPc was started immediately. The decrease in oxygen was measured based on the EPR spectrum and obtained from measurements of peak to peak linewidth as a function of potential oxygen present at 3 minute intervals for 160 minutes in total. The change in linewidth was transformed to oxygen concentration using the predetermined calibration curve presented in supporting information (S3 Fig), which was established prior to the experimental run to account for barometric pressure at that time. From this, the slope of the decrease in oxygen concentration versus time yielded the oxygen consumption rate of the spheroids. The initial oxygen concentration was calculated based on Henry’s Law constant, and taken as 201*μ*M oxygen at 19°C in pure air.

### Statistical Analysis

The data has been presented as mean values ± SD, with *n* denoting the number of replicates per experiment unless otherwise indicated. Comparison between groups were analysed by Student’s t-test (Media versus DPBS/water). Results were analysed using the Friedman non-parametric test due to non-normal data (*n* = 3). A value of *p* < 0.05 was considered significant.

## Results and Discussion

Whilst there are a variety of methods available for determination of oxygen within spheroids, historically it has been acknowledged that the most commonly used method (e.g. micro-electrodes) suffer from several inherent limitations leading to unreliable results (oxygen production/consumption by electrode, signal drift, media requirements etc. [23]). Electron paramagnetic resonance (EPR) oximetry is widely accepted as one of the most reliable techniques with which to measure free radicals and oxygen in tissues [32]. However, despite the acknowledged reliability of this approach, EPR has not been applied to 3D culture systems until now to quantify oxygen gradients. One advantage which EPR based methodology has over normal micro-electrode measurements is that linewidth of spectra is based on pure physical interaction between paramagnetic probe molecules and oxygen within the biological system [27]. In our study, the application of EPR from concept to application (Fig 1) was established firstly with the successful formation of the spheroid around paramagnetic probes with the assistance of an orbital shaker and subsequently with the strength of the signal achieved over time (S2 Fig). Although results are not presented herein, our method was also shown to be applicable to a well characterised tumour based liver spheroid model (i.e HepG2 cell line).

Oxygen micro-environments are known to form within tumour spheroid models as a function of culture time and initial seeding densities of cells and spheroid size [46]. Furthermore, it is widely accepted that tumour cells exhibit greater heterogeneity due to gradual genetic changes as a function of cellular division [47]. Our study provides for the first time evidence of these micro-environments in the non-tumour spheroid model of the rainbow trout gonad cell line RTG-2 over a variety of different spheroid sizes. Previous studies have initiated spheroid experiments (eg. exposure) on day 7 (in both aquatic and mammalian based spheroids), due to a change in cellular structure from loose aggregate to spheroid [2, 11]. This trend was further reiterated in our model, with support for the use of smaller spheroids. EPR spectra were recorded at two time points several days apart in order to encompass normal *in vivo* study durations and spheroid formation. Repeat measures of the same spheroid was possible when aseptic technique was employed during handling, and was confirmed throughout the duration of the study with significant differences recorded between time points and size of spheroids (*p* < 0.05, *n* = 3). The ability of the probe to form sharp defined spectra encased within the spheroid and without agitation allows the identification of oxygen distribution within the model, with repeat recording of spectra entirely possible, as demonstrated during this study. This ability to repeat measure samples is in direct contrast to other oxygen measurement methods such as micro-electrodes which require the destruction of the sample per measurement.

During the study, spectra linewidth did not decrease below 290 mGauss (mG) in the larger spheroid size class (> 700*μ*m diameter), an occurrence not replicated in the literature for Lithium phthalocyanine, despite coming quite close to our zero oxygen linewidth of 200 mGauss (mG) in a purely nitrogen environment. A suggested reason for this disparity between recorded values herein and the literature lies in the purity of the paramagnetic probe, with impure probe particulates reporting different mGauss sensitivity, as previously observed [34]. This difference highlights the importance of calibrating each batch of LiPc probe.

In the context of the current study, hypoxic localisation is defined as the area where oxygen may be limited due to tight junction formation, senescent or necrotic cells. Percentage oxygen concentration within the spheroid model was determined by linewidth difference in sample versus completely oxygenated probe in media (*n* = 3) and was observed to narrow as expected with time and decreased oxygen availability (Table 1). In agreement with our results, previous studies have predicted that viability/viable thickness of the spheroid will tend to decrease with the growth/age of the spheroid [5, 22]. Based on this concentration (%), it was possible to determine the viable rim within the spheroid model and from this to calculate the corresponding hypoxic region of the spheroid as a percentage of overall size. In combination with the initial identification of oxygen concentration within the model, the calculation can be further extended to detect specific oxygen gradients where oxygen is limited (r_*l*_) through diffusion difficulties (oxygen partial pressure and Henry’s law Constant at experimental culture conditions) causing senescent zones and as a consequence zones of necrosis can be identified [22]. Teasing apart differences in hypoxic region from differences in oxygen diffusion into the spheroid from the media, it is clear that there is significant overlap in some seeding densities, allowing the identification of necrotic and quiescent zones. Our study identifies the lowest sized spheroid as having the smallest hypoxic zone in addition to the smallest zone of necrosis/senescence. This is in agreement with the literature expectations of minimal, if absent, necrotic zones in smaller size class spheroids of tumorous and non tumorous origin. Although the results are not presented, the absence of necrosis in the smaller spheroid size (2,500 cells/spheroid) was also confirmed via histological staining, as previously reported for tumour based spheroids [48].

Our study highlights the important association between size and the formation of oxygen limited environments (hypoxia) within the spheroid system, a finding which has already been illustrated in the literature for tumour derived spheroid models [49, 50] but nevertheless has remained unknown till now in the non tumour models. The occurrence of this zone of senescence lends support to the suggestion that necrotic cells arising as quiescent cells die as noted by previous studies [51]. When the smallest seeding density in the RTG-2 cell spheroid is examined closely, a decrease in oxygen concentration and diffusion with time is observed, suggesting the the development of a quiescent zone which may with time lead to a zone of necrosis. This hypothesis is supported by the application of previous studies, which address tumour necrosis formation, with measurements for the non-tumour RTG-2 model [22]. Equally, this trend can also be extended to larger sized spheroids, where minimal oxygen concentration is indicative of large areas of hypoxia and necrosis, a trend repeated throughout the literature and highlighting the comparability of this method to existing knowledge. Both methods (EPR and formulae) of determining oxygen concentration within the spheroid model indicate that when studies requiring a lack of necrosis (such as in the fish liver spheroid model) are required, it is best to work in spheroids whose size does not exceed a 100*μ*m in diameter. As a result, this will allow for a more physiologically relevant native tissue environment which would better duplicate the *in vivo* response, allowing for a reduction in the use of animals during preliminary investigative studies.

While our study shows oxygen as a percentage value in order to identify oxygenated 3D models to be used as an *in vitro* alternative to live fish tests, other studies report in terms of partial pressure. Oxygen partial pressure values of 50–60 mm Hg in the spheroid model have been reported in malignant cells using 3D oximetry methods [52]. Apart from the inherent differences between the two cell lines used (i.e. non transformed fish vs transformed mammalian, incubation temperature etc.), it is difficult to make a direct comparison between the two methods as different parameters were used. Furthermore, assumptions made with respect to calculating the partial pressure will require taking into consideration several biological and physiochemical factors (i.e. water vapour pressure, humidity, size of the spheroids, nature of the tight junctions etc.). However, if the study is standardized to expression of percent oxygen partial pressure, then comparison is possible. For example, assuming that the partial pressure of oxygen available at 37°C is 150 mm Hg (assuming high water vapour saturation), then the external value of 120–130 mm Hg would imply that the outer layer is almost completely oxygenated (∼90%) at this temperature. This is logical if the outer layer is limited to 10–20 μm, as in our model.

In addition to numerous benefits of using EPR over other methods, it should also be noted that the inherent sensitivity of the paramagnetic probe (i.e. LiPc) allows the user to report on unusual occurrences within the system, such as the apparent increase in oxygen concentration (∼11–50 *μ*M) with time in the larger sized spheroids (600*μ*m) (Table 1). We have attributed this increase in oxygen to the cells within the centre of the spheroid dying due to lack of nutrients, as indicated by the size of the anoxic zone in both these size classes. While this phenomenon has so far not been reported in spheroid systems prior to this study, the hypothesis is supported by other studies that noted a similar trend of increase in dissolved oxygen level in monolayer cultures which were correlated with an increase in cell death attributed to lack of nutrients [53], but the precise mechanism as yet remains unclear.

**Fig 2:**
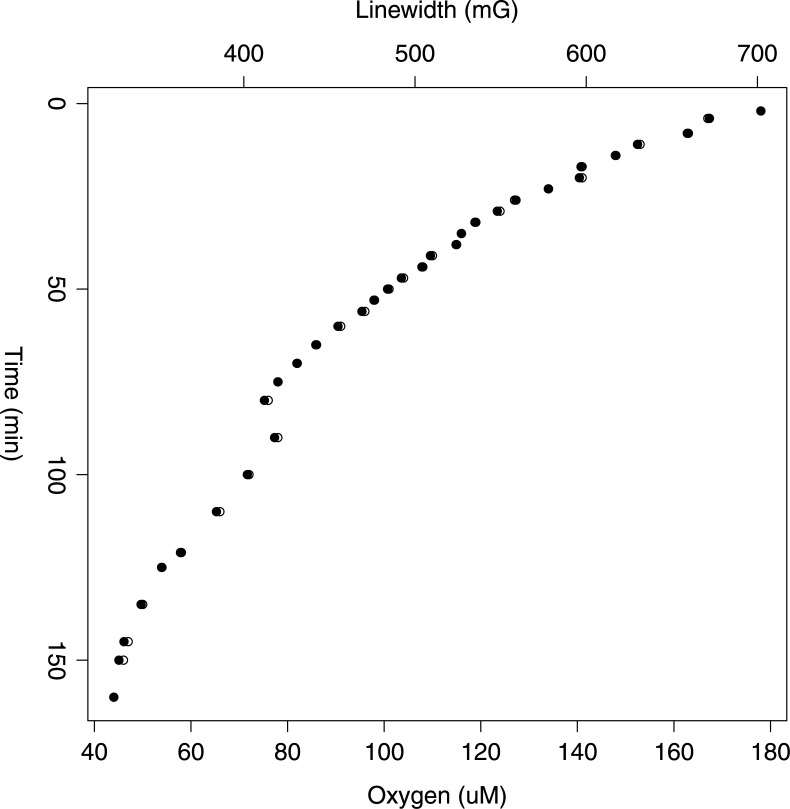
Oxygen consumption rate using EPR for a spheroid of ∼120*μ*m on day 7. A decrease in oxygen concentration relative to time is explained by the formula -2.21T+623.77 (R^2^ = 0.91), where *μ*mole of oxygen is represented by *μ*M and T is in minutes (160 min) and where the linear range occurs within the first 50 minutes. This linear range corresponds to an oxygen concentration range of 178-101*μ*M. It appears that spheroids are unable to consume oxygen in a linear manner below 100 *μ*M, perhaps due to formation of oxygen diffusion gradients. The OCR rate is a result of the average of two separate experimental runs.

In order to better understand the kinetics of oxygen consumption, we preformed oximetry measurements in a sealed environment using LiPc probes. As is clearly demonstrated in Fig 2, oxygen is rapidly consumed by the spheroids in this sealed environment as demonstrated by a rapid decrease in linewidth. Oxygen concentrations (*μ*M) were back-calculated within the model from a standard curve of oxygen with respect to literature reported availability at specific temperature (S3 Fig), and presented on the z-axis as a function of linewidth. The kinetics of oxygen consumption could not be recorded accurately for the first 7–10 min due to the inherent variation of the linewidth. Previous studies have identified that in biological tissues, a part of the oxygen sensitive LiPc form is converted into an oxygen-insensitive form [54]. Thus, it is likely that this may have occurred here due to the initial broadening/variable spectra during OCR recording. Despite this, the study method is not devalued in anyway due to the acknowledgement of the initial variable spectra being a common phenomenon associated with the measurement of oxygen concentrations using this probe. Prior studies have suggested that it is simply related to the size of the particulate used, and to increase spectra strength, fine tuning of paramagnetic particulate size must be employed for the specific model under investigation [45, 54]. In addition, reported values in the literature highlight the importance of calibrating all batches of paramagnetic probe within experimental confines. In order to limit variation, a stock solution of LiPc particulates was prepared prior to the experiment and used throughout the study limiting variability between separate recordings.

### Summary and conclusion

It is widely accepted that the biological activities present in spheroids more closely reflect key characteristics of the living organism, and as such may offer a more relevant alternative to *in vitro* exposure in biological research. Previous studies have theorized optimal spheroid diameter in non-tumour mammalian spheroids which allows for effective diffusion of oxygen through the spheroid based on cell viability and functionality [5, 55]. However, these methods are destructive, do not allow for repeat measures, and do not answer the question about whether a compound (of similar size) can actually diffuse through the tight junctions prevalent during spheroid formation/maintenance. Our study addresses this question non-destructively and supports the use of these *in vitro* studies as a tool to aid reduction in whole animal studies. In addition, our study also addresses the size range which is appropriate to use in non-tumour studies to ensure minimal micro-environment formation. By adjusting the size of the spheroid (<150 *μ*m diameter), it is possible to limit the percentage of the spheroid which is hypoxic/necrotic (<2 % allows for presence of senescent cells), a finding in line with previous studies [5]. Although tissue origin in the previous studies differs from our model (murine liver versus fish cell line), suggestions of no oxygen limitation in spheroids below 100*μ*m are in complete agreement. In conclusion, EPR has provided insights into the size and cell seeding densities at which oxygen gradients will play a confounding role in subsequent exposure applications and thus enable the wider use of the spheroid model to non-tumour based biological studies.

## Supporting Information

S1 Fig Spheroid with centrally located LiPc particles. The probes are clearly visible centrally, even in small spheroids (2,500 cells/spheroid). Scale bar represents 100 *μ*m in all images bar (a). In this instance, this scale bar is set at 50*μ*m.
(PDF)

S2 Fig EPR Linewidth of LiPc probe in RTG-2 spheroid. EPR spectra of the smallest spheroid in the study (2,500 cells/spheroid) at 2 time points with LiPc probes centrally located reporting on oxygen within. Oxygen is quantified based on a decrease in spectrum linewidth with decreased oxygen availability relative to a control (100% oxygenated). Arrows indicate a broadening or narrowing of linewidth relative to oxygen availability in the spheroid system.
(EPS)

S3 Fig Calibration curve of LiPc probe in varying oxygen concentrations. Oxygen concentrations (*μ*M) were obtained from literature based on known solubility of oxygen with respect to temperature in kelvin. The relationship between the two is described by 202.94*LW*+2.65 where *R*^2^ = 0.99.
(EPS)
