# Correction: Direct Measurements of Oxygen Gradients in Spheroid Culture System Using Electron Parametric Resonance Oximetry

**DOI:** 10.1371/journal.pone.0160795

**Published:** 2016-08-02

**Authors:** Laura M. Langan, Nicholas J. F. Dodd, Stewart F. Owen, Wendy M. Purcell, Simon K. Jackson, Awadhesh N. Jha

In the title of this article, the word “Parametric” should be “Paramagnetic.” The correct title is: Direct Measurements of Oxygen Gradients in Spheroid Culture System Using Electron Paramagnetic Resonance Oximetry. The correct citation is: Langan LM, Dodd NJF, Owen SF, Purcell WM, Jackson SK, Jha AN (2016) Direct Measurements of Oxygen Gradients in Spheroid Culture System Using Electron Paramagnetic Resonance Oximetry. PLoS ONE 11(2): e0149492. doi:10.1371/journal.pone.0149492
