# Supplementary figures and images for: Direct Measurements of Oxygen Gradients in Spheroid Culture System Using Electron Parametric Resonance Oximetry
Source: PLoS One. 2016 Feb 22;11(2):e0149492. doi: 10.1371/journal.pone.0149492 (PMC4764677; doi:10.1371/journal.pone.0149492)

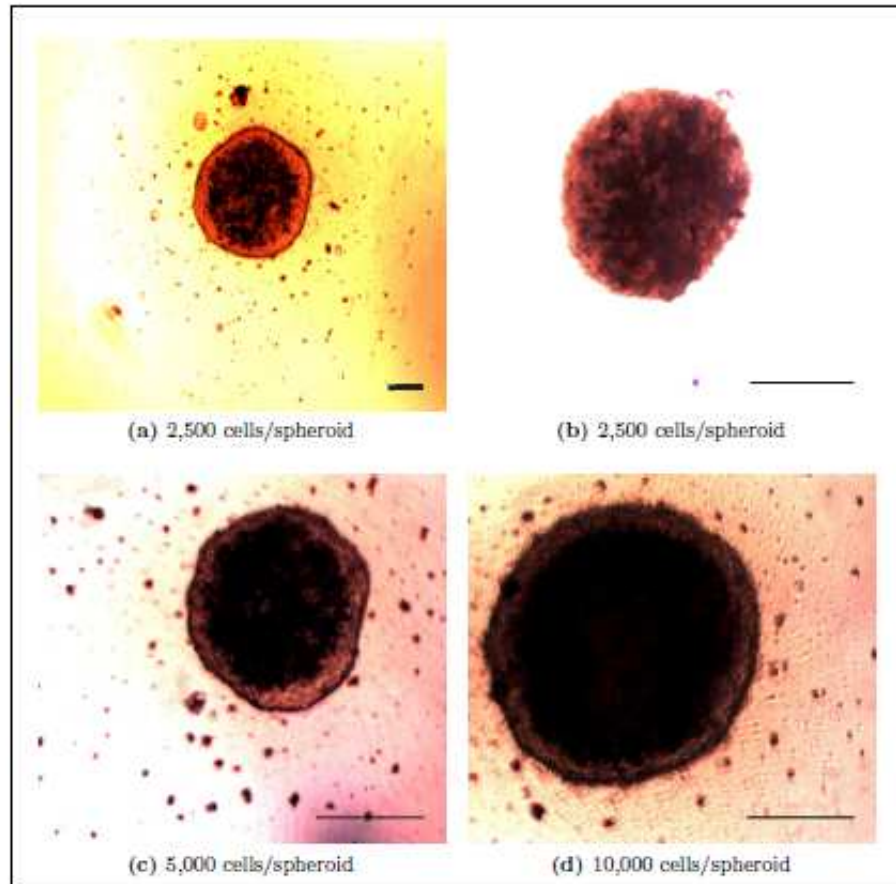

Supplement: S1 Fig — The probes are clearly visible centrally, even in small spheroids (2,500 cells/spheroid). Scale bar represents 100 μm in all images bar (a). In this instance, this scale bar is set at 50μm. (PDF) [file pone.0149492.s001.pdf]
